# Impact of Dapagliflozin Adjunctive Therapy on the Progression of Chronic Kidney Disease in Patients with Type 2 Diabetes and Chronic Kidney Disease Stages 2–5: A systematic review and meta-analysis

**DOI:** 10.18295/squmj.12.2023.091

**Published:** 2024-08-29

**Authors:** M. Kanimozhi, Manisha Bisht, Sikha Morang, Surabhi Thapliyal, Manbir S. Bassan, Shailendra Handu

**Affiliations:** Department of Pharmacology, All India Institute of Medical Sciences, Rishikesh, India

**Keywords:** Chronic Kidney Disease, Dapagliflozin, Glomerular Filtration Rate, SGLT2 Inhibitors, Type 2 Diabetes Mellitus, Albuminuria

## Abstract

This meta-analysis investigated efficacy of dapagliflozin as adjunctive therapy for patients with type 2 diabetes mellitus (T2DM) and chronic kidney disease (CKD) stages 2–5. A systematic search was conducted of selected databases for randomised controlled trials that reported the mean change in estimated glomerular filtration rate (eGFR) and urine albumin-creatinine ratio (UACR) from baseline. Out of 1,682 identified studies, 9 trials comprising 13,057 patients were included. A pooled estimate of 5 studies indicated that dapagliflozin did not affect eGFR; however, in 2 studies, it significantly reduced chronic eGFR decline compared to placebo (mean difference [MD] ± 2.74; 95% confidence interval [CI]: 1.55, 3.92; *P* <0.00001). Additionally, a pooled estimate of 4 studies showed that dapagliflozin significantly reduced UACR (MD −23.99%; 95% CI: −34.82–−13.15; *P* <0.0001; I^2^ = 0%). Therefore, long-term use of dapagliflozin significantly attenuates eGFR decline and reduces albuminuria in patients with T2DM and CKD.

Chronic kidney disease (CKD) is a progressive condition characterised by a gradual decline in renal function, eventually leading to end-stage renal disease (ESRD) or renal failure. Currently, nearly 12% of the world’s population is affected by CKD, and its prevalence is increasing.^1^

Approximately two-thirds of CKD cases are attributable to diabetes and hypertension, with glomerulonephritis, autoimmune diseases and age-related kidney conditions accounting for the remainder.^2^ The term diabetic kidney disease (DKD) is used when CKD results from diabetic microvascular complications, while non-diabetic kidney disease (NDKD) refers to CKD from other causes.

Patients with diabetes may also have CKD from non-diabetic causes, resulting in NDKD. Only a renal biopsy can provide a definitive aetiology for CKD; however, kidney biopsy is not feasible in routine clinical practice. Additionally, hyperglycaemia may hasten the progression of CKD in both DKD and NDKD patients and raise their risk of cardiovascular disease (CVD).^2^ Consequently, the primary therapeutic objective in patients with diabetes and CKD (either DKD or NDKD) is to prevent CKD progression and reduce CVD risk.

Substantial research has focused on a novel family of anti-diabetic drugs called sodium-glucose co-transporter-2 (SGLT2) inhibitors, particularly dapagliflozin, which demonstrated considerable reno-protective effects in the Dapagliflozin and Prevention of Adverse Outcomes in Chronic Kidney Disease (DAPA-CKD) trial. Based on the findings from this trial, dapagliflozin was licensed in 2021 for the management of CKD to lower adverse renal events and improve CVD outcomes in patients with and without type 2 diabetes mellitus (T2DM).^1^ However, no summary estimate of its renal efficacy in patients with CKD (stage 2–5) and T2DM has been reported so far.

Estimated glomerular filtration rate (eGFR) and urine albumin-creatinine ratio (UACR) are extensively used as surrogate endpoints in clinical settings to measure CKD progression.^3^ The combination of a drop in eGFR and an increase in UACR is significantly associated with a higher risk of CKD progression than either marker alone. Therefore, dapagliflozin's reno-protective effects can be effectively documented by evaluating the mean changes in eGFR and UACR from baseline.

This systematic review and meta-analysis aimed to estimate the impact of dapagliflozin adjunctive therapy on the progression of CKD (measured in terms of mean changes in eGFR and UACR from baseline) in individuals with T2DM, to generate sufficient scientific evidence for its clinical use.

## Methods

This systematic review and meta-analysis adhered to the Preferred Reporting Items for Systematic Reviews and Meta-Analysis (PRISMA) criteria.^4^ The protocol was registered in the International Prospective Register of Systematic Reviews (PROSPERO) and is accessible via the PROSPERO website (CRD42022304631).

### DATA SOURCES AND SEARCH

Electronic databases such as PubMed, Scopus, Cochrane and Ovid were searched for relevant studies published from February 2000 to November 2022. Additional searches to identify eligible studies were conducted in the Clinical Trials Registry of India and ClinicalTrials.gov, as well as through manual searches.

Medical subject headings (MeSH) terms such as ‘dapagliflozin’ AND ‘CKD’; ‘dapagliflozin’ AND ‘chronic kidney disease’ AND ‘type 2 diabetes’; ‘dapagliflozin’ AND ‘albuminuria’ AND ‘eGFR’ were used to search for relevant studies. The search results were further refined using filters for full-text and English-language articles.

Before submission, another electronic database search was conducted and a final analysis report was compiled to ensure that recent updates were included. A summary of the electronic database search is provided in the supplementary tables [Supplementary Tables 1 & 2].

### ELIGIBILITY CRITERIA

Randomised controlled trials (RCTs) and post hoc analyses of RCTs were included if they met the following criteria: (i) conducted on patients with T2DM and CKD stages 2–5 of any aetiology (baseline eGFR <90 mL/min/1.73 m^2^); (ii) used dapagliflozin 10 mg once daily, which is the most commonly prescribed dosage in clinical practice for the treatment of CKD, as an interventional drug adjunct to the standard of care (SOC); (iii) compared dapagliflozin to either placebo or any other oral hypoglycaemic agents (OHAs)/anti-CKD drugs; (iv) conducted for ≥12 weeks (since the stabilisation period of dapagliflozin’s effects on metabolic and renal parameters takes at least 8–12 weeks); and (v) assessed renal endpoints such as mean change in eGFR and UACR.

The following studies were excluded: studies other than RCTs (non-randomised controlled trials, case report, case series, cross-sectional studies, cohort studies); studies conducted on type 1 diabetes and CKD stage 1 (KDIGO) patients (baseline eGFR >90 mL/min/1.73 m^2^) as well as the non-diabetic population; studies that used dapagliflozin 5 mg or a fixed-dose combination of dapagliflozin as the intervention; single-arm studies; studies conducted for <12 weeks; and studies that did not assess the desired renal outcomes.

### STUDY SELECTION

Relevant studies identified from the aforementioned databases were exported to the citation manager (Zotero; Corporation for Digital Scholarship, Virginia, USA) for duplicate removal. Afterwards, all individual papers were examined by 2 independent authors for eligibility according to the criteria, first by title and abstracts, then by full text in cases of uncertainty. In case of discrepancies between the 2 authors, a third independent author made the final decision.

### DATA EXTRACTION

Data were extracted to assess the following primary outcomes: mean change in eGFR and mean percentage change in UACR from baseline in both interventional and control groups. Prevention of CKD progression was defined as an increase in mean eGFR or a reduction in the decline in eGFR and a decrease in the mean percentage UACR from baseline.

From the eligible studies, information such as study design, study duration, median follow-up duration, interventional drug used, comparator drug used, sample size and other outcome-related data were extracted. For post hoc analyses, primary trials were used to obtain additional details beyond those presented in the post hoc papers. WebPlotDigitizer (Automeris LLC, Texas, USA) was used to extract data from graphs and pictorial representations. Data extraction was primarily and independently performed by 2 authors (MK and SM) and cross-verified by a third author (MB).

### QUALITY ASSESSMENT

A qualitative assessment of the included papers was conducted using Cochrane’s risk-of-bias assessment tool for RCTs (RoB 2; Cochrane Methods Group, London, UK). The domains used to assess the risk of bias were: the randomisation process, deviation from the intended interventions, missing outcome data, measurement of outcome and selection of the reported results. Based on the assessments made according to these domains, the included papers were categorised into either low risk, some concerns or high risk. Quality assessment was conducted by 2 independent authors (MK and ST) and cross-verified by a third author (MB).

### DATA SYNTHESIS AND ANALYSIS

A meta-analysis was performed to quantitatively assess the outcomes of the included studies using Review Manager software, Version 5.4 (RevMan International, New York, USA). The heterogeneity between the studies was estimated using the I^2^ test. An I^2^ value >50% was interpreted as moderate to high heterogeneity, while a value <50% was considered low to moderate heterogeneity. To pool the data from the included studies, the random effects model was utilised, and the mean difference (MD) or standardised mean difference (SMD), along with its corresponding 95% confidence interval (CI), of the desired outcomes was calculated between the 2 groups to measure the treatment effect precisely.

After reviewing the initial results of one of the primary outcomes—mean change in eGFR from baseline—the authors conducted a non-prespecified subgroup analysis to compare the mean change in chronic eGFR slope from 2 trials between dapagliflozin and a placebo. For this analysis, the authors calculated the MD and related 95% CI using the random effects model.

### QUALITY OF EVIDENCE

The strength of evidence of the meta-analysis results was assessed using the GRADEpro (Evidence Prime, Ontario, Canada) software according to the following criteria: risk of bias, inconsistency, imprecision, indirectness and other considerations such as publication bias.^5^ Based on these criteria, the quality of evidence was graded as high, moderate, low or very low.

## Results

A total of 1,681 records were identified (PubMed: 324, Scopus: 580, Ovid: 491, Cochrane registry: 286) from the initial electronic database search. Approximately 869 duplicate papers were excluded with the assistance of the citation manager (Zotero) and 488 irrelevant studies were removed using manual filters. For the remaining 219 records, a screening based on title and abstract was conducted by 2 individual authors, resulting in the removal of 140 non-RCTs.

**Figure 1:**
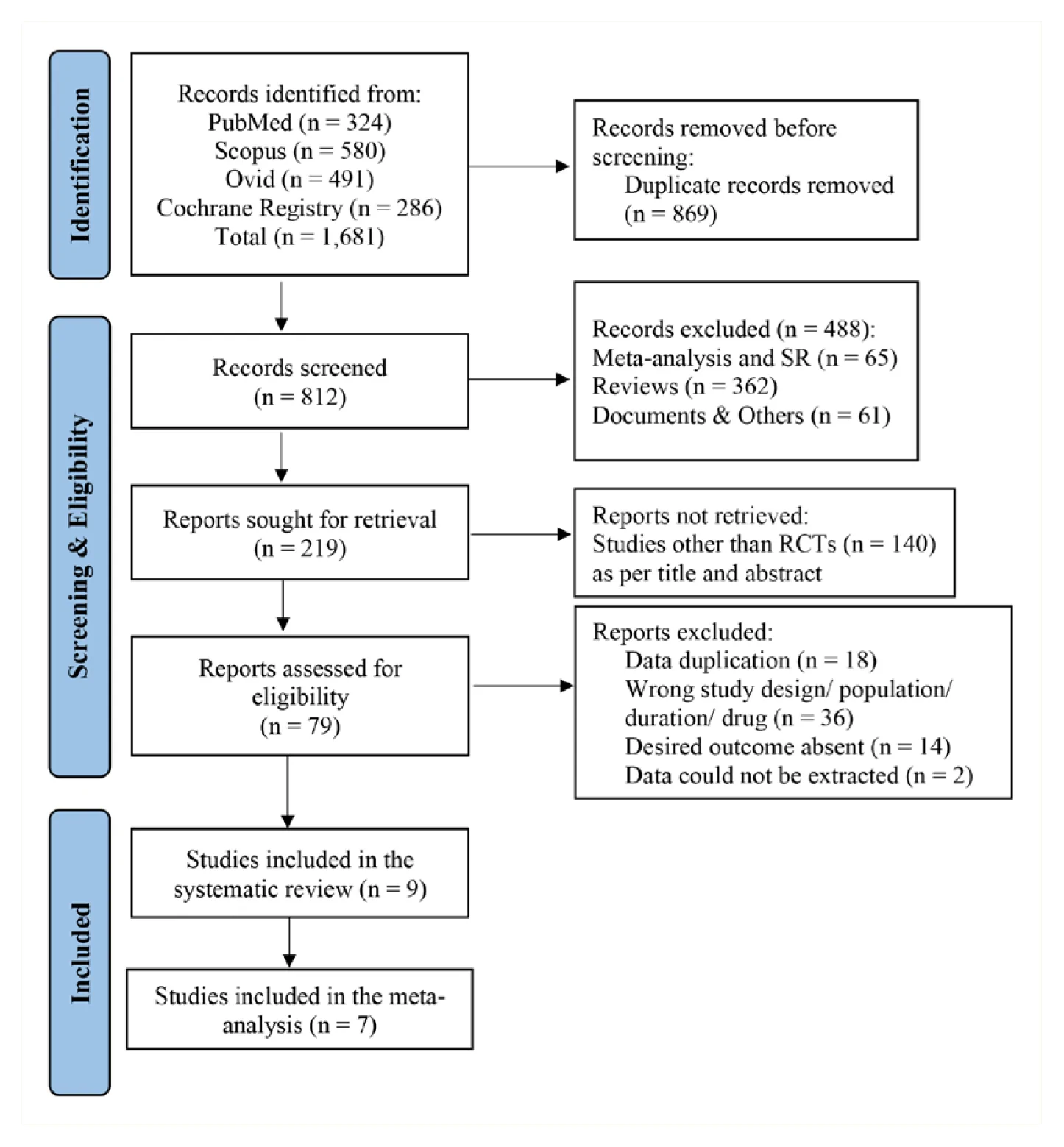
Flow diagram showing how the studies were identified in the current systematic review. *RCT = randomized controlled trial*.

Finally, 79 full-text papers were examined for adherence to the current study’s eligibility criteria. Among them, 9 studies (including 13,057 participants) were included in the systematic review, and 7 (representing 4,713 participants) were retained for meta-analysis [Figure 1]. The reasons for the exclusion of full-text articles are provided in Supplementary Table 1.

### BASELINE CHARACTERISTICS OF STUDIES INCLUDED

The studies considered in this systematic review and meta-analysis were published before November 2022. Among the 9 analysed studies, 6 were RCTs, 1 was a post hoc study and 2 were secondary exploratory analyses. The included studies had 13,057 participants with T2DM and CKD (eGFR <90 mL/min/1.73 m^2^). All 9 studies had dapagliflozin 10 mg once daily as their primary intervention, along with a background SOC, and 8 studies had placebo as their comparator; 1 study had valsartan 80 mg as its comparator drug. The maximum study duration/median follow-up duration among the included studies was 4 years, and the minimum was 3 months. Dapagliflozin's effect as an adjuvant to SOC on CKD prognostic biomarkers such as eGFR and UACR was assessed in these included studies. The baseline demographic details of the evaluated studies are summarised in Table 1.

### RISK-OF-BIAS OF THE ASSESSED STUDIES

**Figure 2:**
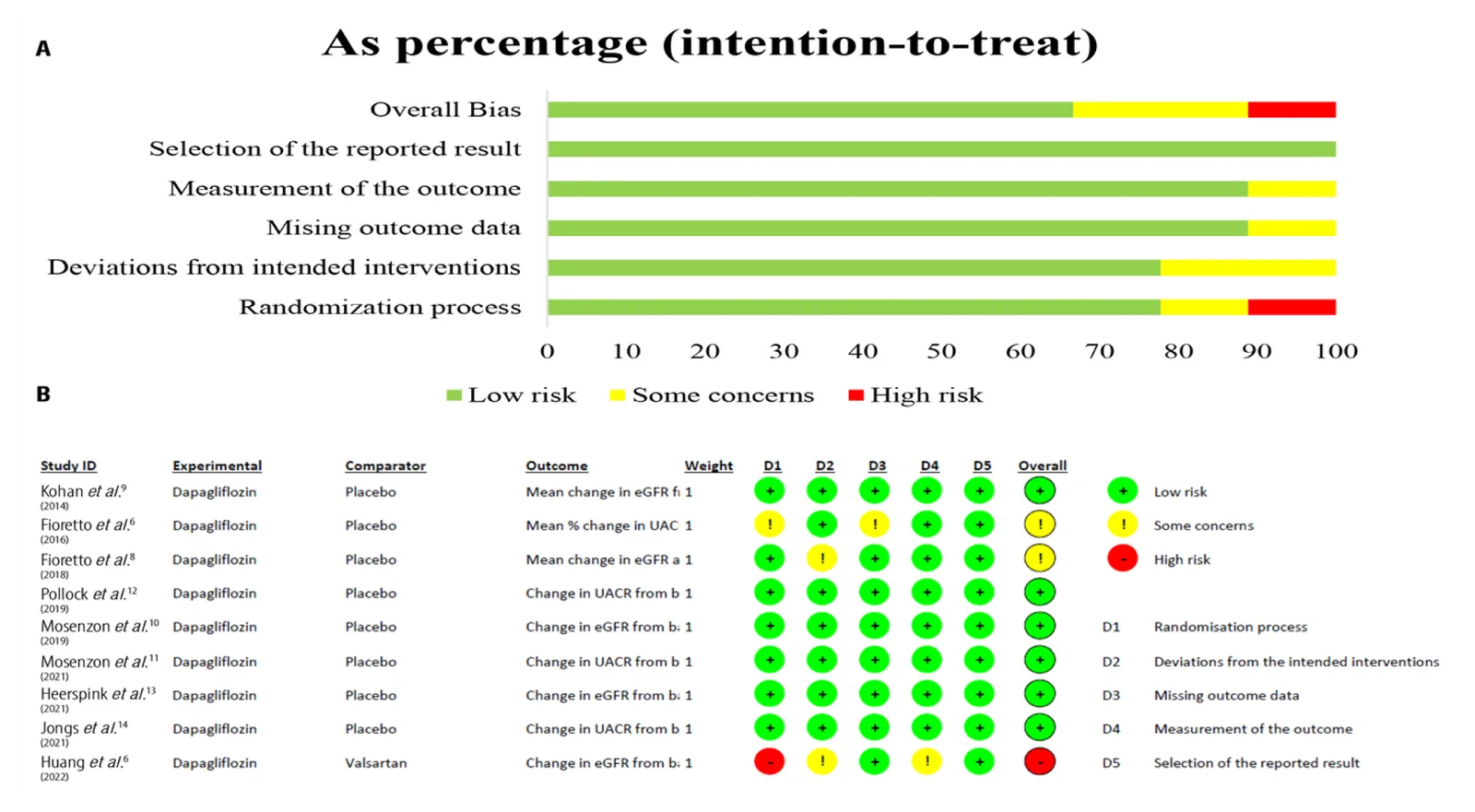
**A:** Risk-of-bias assessment graph of the included studies.^6^–^14^ **B:** Overall risk-of-bias assessment. *UACR = urine albumin-creatinine ratio; eGFR = estimated glomerular filtration rate*.

Among the 9 included studies, 1 had a high overall risk of bias as no mention of the methods used for randomisation was provided.^6^ Two studies had a moderate risk of bias due to concerns about missing outcome data and deviation from intended interventions.^7^^,^^8^ Moreover, 6 studies had an overall low risk of bias [Figure 2].

### SYSTEMATIC REVIEW

A summary of dapagliflozin’s effect as an adjunct to SOC on eGFR and UACR in patients with T2DM and CKD (eGFR <90 mL/min/1.73 m^2^), as predicted in individual studies [Table 2].^6^–^14^

The results of the included studies showed that short-term dapagliflozin use did not affect eGFR significantly, but its chronic use prevented a great decline in the eGFR slope.^8^^,^^10^^,^^12^^,^^13^ Additionally, dapagliflozin use was associated with a significant reduction in mean percentage UACR from baseline. Therefore, dapagliflozin prevents CKD progression in T2DM patients with a baseline eGFR <90 mL/min/1.73 m^2^.

### META-ANALYSIS

**Figure 3:**
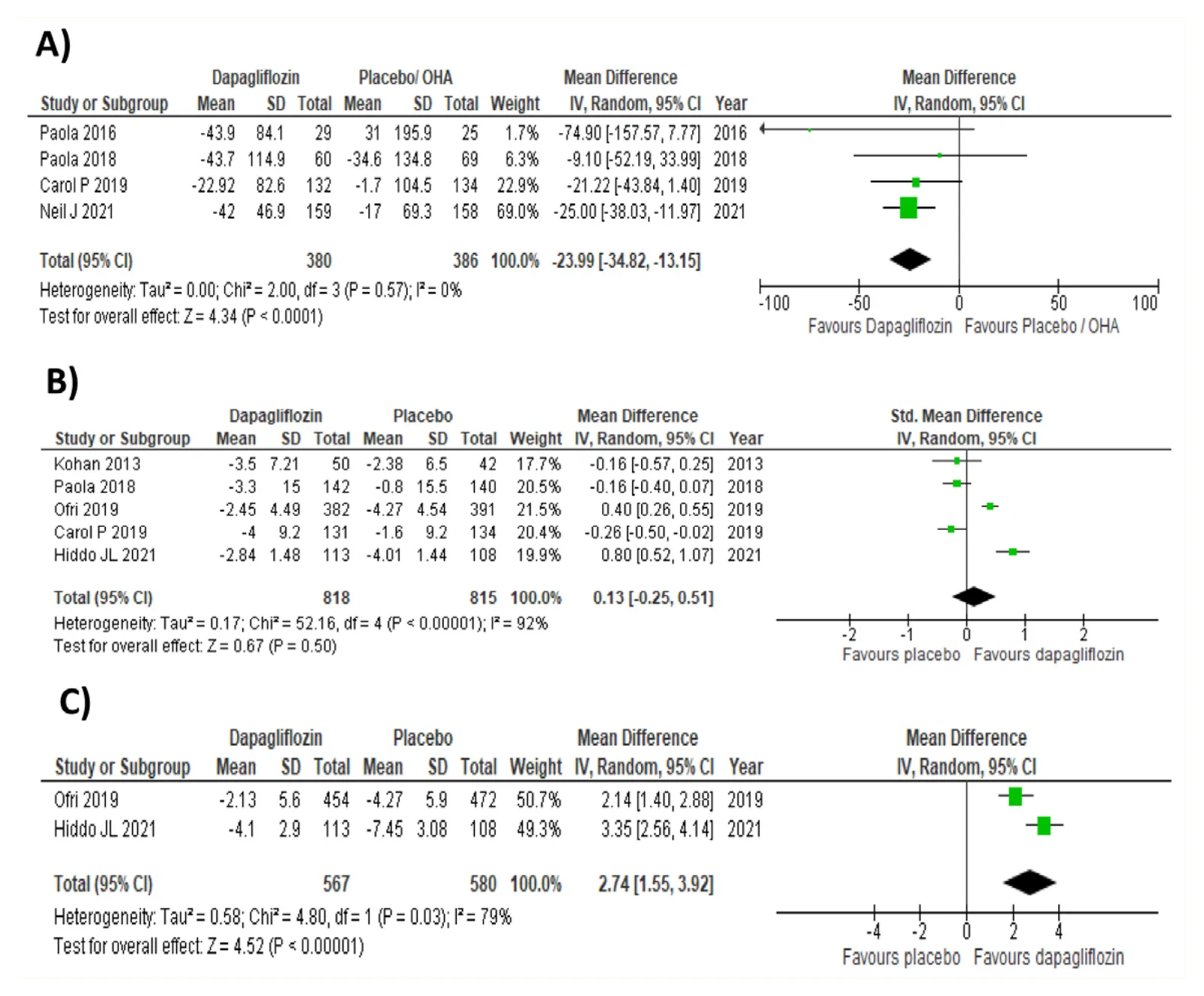
Forest plots showing **(A)** the mean percentage change in urine albumin-creatinine ratio from baseline, **(B)** the mean change in estimated glomerular filtration rate from baseline–total slope (ml/min/1.73 m^2^), and **(C)** the mean change in chronic estimated glomerular filtration rate slope (ml/min/1.73 m^2^). *SD = standard deviation; OHA = oral hypoglycaemic agents; CI = confidence interval*.

A meta-analysis was executed for 7 of the 9 included studies [Figure 3]. Among the 7 studies, 5 reported results for mean change in eGFR, and 4 showed results a mean percent reduction in UACR from baseline.

### MEAN CHANGE IN eGFR FROM BASELINE

Five studies, including 818 individuals in the dapagliflozin group and 815 patients in the placebo group, were quantitatively assessed for mean changes in eGFR from baseline values. Using the random effects model, the pooled estimate of the 5 studies was determined, showing an SMD of +0.13 mL/min/1.73 m^2^ (95% CI: −0.25–0.51; *P* = 0.50; I^2^ = 92%, *P* <0.0001) between the two groups. This implies that, compared to placebo, dapagliflozin as an adjunct to SOC is not associated with a statistically significant rise in eGFR values from baseline.

The obtained I^2^ value of 92% indicates that the included studies were statistically highly heterogenous and the effect was inconsistent across the studies. To determine the stability of the current study’s results, the authors conducted a sensitivity analysis (excluding short-duration studies), which showed an SMD of + 0.38 mL/min/1.73 m^2^ (95% CI: −0.04–0.79; *P* = 0.08; I^2^ = 87%, *P* = 0.0005) between the two groups. This result also confirmed the statistically insignificant effect of dapagliflozin on the total slope of eGFR compared to placebo in longer duration studies.

### MEAN CHANGE IN CHRONIC eGFRSLOPE (SUB-GROUP ANALYSIS)

To estimate the chronic treatment effect of dapagliflozin, the authors further analysed the chronic eGFR slope between 1 to 3 years from two studies using the random effects model.10,13 This analysis yielded an MD of +2.74 mL/min/1.73 m^2^ (95% CI: 1.55–3.92; *P* <0.00001; I = 79%, *P* = 0.03) between the two groups, indicating that chronic dapagliflozin use caused a more significant attenuation of eGFR decline compared to placebo. Kohan *et al*. conducted a long-duration study (104 weeks), but their results were not included in this analysis due to difficulties in data extraction.^9^

### MEAN PERCENTAGE CHANGE IN UACR FROM BASELINE

Four studies, with 380 participants in the dapagliflozin group and 386 individuals in the placebo group, were quantitatively assessed for their mean percentage reduction in UACR values from baseline. Using the random effects model, the pooled estimate of the 4 studies revealed an MD of −23.99% (95% CI: −34.82–−13.15; *P* <0.0001; I^2^ = 0%) between the two groups. The I^2^ value was 0%, indicating that all the analysed studies were statistically homogenous. This confirms that the use of dapagliflozin as an adjunct to SOC reduces UACR in a statistically significant manner compared to placebo.

### QUALITY OF EVIDENCE

The GRADEPro (Evidence Prime) software was used to grade the quality of evidence of the results obtained during the meta-analysis in the current study [Supplementary Figure 1]. Accordingly, the results for mean change in UACR from baseline were found to have a high quality of evidence, suggesting that future research is unlikely to change the current study’s effect estimate. Conversely, the results for the mean change in eGFR from baseline had a low quality of evidence, implying that future research is more likely to change the current study’s effect estimate. Finally, the results for the mean change in chronic eGFR slope had a moderate quality of evidence, suggesting that future research might change the current study’s effect estimate.^15^

## Discussion

SGLT2 inhibitors are a unique class of oral anti-hyperglycaemic agents approved for the treatment of T2DM, both as monotherapy and as an add-on to standard anti-diabetic care. SGLT2 inhibitors exert their anti-diabetic effect by inhibiting the reabsorption of glucose by the SGLT2 channels present in the proximal renal tubular cells, resulting in glycosuria. This glycosuria is associated with significant glucose-induced osmotic diuresis and natriuresis, which lead to renal haemodynamic changes such as the activation of tubuloglomerular feedback and afferent arteriolar constriction.^16^ These hemodynamic changes manifest clinically as acute eGFR reduction and may sometimes result in acute kidney injury.^17^ Since the primary action of SGLT2 inhibitors is on the proximal renal tubular cells, their glycaemic efficacy decreases with worsening renal function, but their reno-protective effects become more prominent as renal impairment advances.^18^

Dapagliflozin, a highly effective and selective SGLT2 inhibitor, showed promising reno-protective effects in the DAPA-CKD trial.^19^ However, the Food and Drug Administration has issued a warning regarding the greater probability of developing acute kidney injury with its use.^20^ Most of the clinical trials that documented dapagliflozin’s reno-protective effects were conducted in both diabetic and non-diabetic populations across different stages of CKD (KDIGO 1–5) and even in individuals with normal kidney function. The renal composite outcome (i.e., a sustained decline in eGFR >40% or >50%, progression to ESRD, cardiovascular death or renal death) was the primary endpoint in most of the trials assessed in this study, and very few of these trials assessed dapagliflozin’s direct effect on eGFR slope in patients with T2DM and CKD.

Therefore, intending to quantify the effect size, the authors estimated the impact of dapagliflozin adjunctive therapy on CKD progression in people with T2DM and CKD stages 2–5 (eGFR <90 mL/min/1.73 m^2)^. To estimate this effect, the authors chose two independent prognostic biomarkers of CKD progression—eGFR and UACR.^21^^,^^22^ These 2 prognostic biomarkers are inexpensive, widely available and more accurate predictors of renal function when used in combination rather than alone.^23^ The authors selected dapagliflozin 10 mg once daily as the intervention because it is the most prescribed dosage in routine clinical practice.

Dapagliflozin, like other SGLT2 inhibitors, may reduce glomerular filtration pressure, resulting in a decrease in UACR.^23^ It is evident from all the included trials that dapagliflozin’s use as an adjunct to SOC is associated with a significant reduction in UACR, indicating that it improves albuminuria and helps halt the progression of CKD. The meta-analysis results further confirmed that, compared to placebo, dapagliflozin significantly decreases UACR.

Regarding the mean change in eGFR, the meta-analysis results showed highly inconsistency across the included studies (I^2^ = 92%). This variability is likely due to the differences in the populations studied (e.g. Huang *et al*. only studied patients with diabetic nephropathy) and the fact that shorter duration studies were also included (Fioretto *et al*.; Pollock *et al*.).^6^^,^^8^^,^^12^

Although 3 studies had longer durations and reported almost identical mean baseline eGFR values for the participants, their results varied.^9^^,^^10^^,^^13^ This discrepancy might be attributed to differences in the proportion of participants across various eGFR subgroups, mean age (68 years in Kohan *et al*.’s study compared to 64.1 years in Heerspink *et al*.’s study), mean HbA1c, mean body weight of the participants the different formulae used for calculating eGFR (MDRD in Kohan *et al*.: affected by race; CKD-EPI in Heerspink *et al*.: preferred for diabetic patients) and variations in the SOC administered.^24^

Additionally, the pooled estimate results of the current study might have been insignificant due to the initial acute eGFR reduction associated with dapagliflozin use, which was reported in nearly all the included studies. Similar to other SGLT2 inhibitors, dapagliflozin also triggers activation of the tubuloglomerular feedback mechanism, leading to hypovolaemia and potentially precipitating acute pre-renal failure.^25^ However, the meta-analysis results of the current study clearly demonstrate that dapagliflozin has a positive effect on eGFR preservation, which remains clinically meaningful.^26^^,^^27^ The estimation of chronic eGFR slope observed in 2 studies also revealed that dapagliflozin use was associated with significantly lesser decline in eGFR over time compared to placebo, confirming that the insignificant result was likely due to the initial acute eGFR reduction.

This study has several limitations. There was high heterogeneity among the included studies regarding the mean change in eGFR from baseline. Additionally, the study relied on secondary, exploratory or safety endpoints. There were also discrepancies in the standard background care provided in the included studies and the exclusion of articles written in languages other than English. Due to difficulties in data extraction, a subgroup analysis among distinct eGFR and UACR groups could not be performed.

It is well known that patient factors such as age, gender, ethnicity, co-morbidities and background medications can influence net effect estimates.^28^ However, due to data extraction difficulties, a sensitivity analysis incorporating these factors as co-variates could not be conducted for the net effect estimates of both eGFR and UACR.

## Conclusion

This study concluded that dapagliflozin, when used as an adjunct to SOC, is associated with a significantly lesser decline in eGFR and a reduction in albuminuria progression in patients with T2DM and CKD stages 2–5. Both eGFR and UACR are independent prognostic predictors of CKD progression, and dapagliflozin’s beneficial effects on both biomarkers confirm its reno-protective properties. Given that these conclusions are based on a limited number of studies, future research involving a larger number of trials is needed to validate these findings.

## Supplementary Information



## Figures and Tables

**Table 1:** Baseline demographic details from the included studies^6^–^14^

Author and publication year	Study design	Intervention	Comparator	Standard/background care	Study duration and follow-up	Number of participants	Baseline eGFR (MDRD) and UACR for inclusion	Mean age ± SD in the dapagliflozin group	Mean baseline eGFR (mL/min/1.73 m^2^) mean ± SD	Mean baseline UACR (mg/g) median (range)	Outcome assessed	Included in SR/MA
Kohan *et al*.^9^ (2014)	Randomised, double-blind, multicentric, placebo-controlled trial	Dapagliflozin 10 mg once daily and 5 mg once daily	Placebo	Standard pre-enrolment anti-diabetic regimen given	Study duration: 104 weeks	Total: 252 Dapagliflozin 10 mg: 85 Placebo: 84	eGFR: 30–60 mL/min/1.73 m^2^ (MDRD)	68 ± 7.7 years	Dapagliflozin: 43.9 ± 10.6 Placebo: 45.6 ± 10.0	Placebo: 67 (20–367) Dapagliflozin: 73 (9–352)	Change in eGFR from baseline at week 104	SR and MA
Fioretto *et al*.^7^ (2016)	Post-hoc analysis of Kohan *et al*.	Dapagliflozin 10 mg OD and 5 mg OD	Placebo	Standard pre-enrolment anti-diabetic regimen given	Study duration: 104 weeks	Total: 166 Dapagliflozin 10 mg: 56 Placebo: 57	eGFR: 30–60 mL/min/1.73 m^2^ (MDRD UACR: ≥30 mg/g	68 ± 7.7 years	Dapagliflozin: 43.9 ± 10.6 Placebo: 45.6 ± 10.0	Placebo: 67 (20–367) Dapagliflozin: 73 (9–352)	Change in UACR from baseline at 104 weeks	SR and MA
Fioretto *et al*.^8^ (2018)	Randomised, double-blind, parallel group, placebo-controlled study	Dapagliflozin 10 mg OD	Placebo	Standard pre-enrolment anti-diabetic regimen given	Study duration: 24 weeks	Total: 321 Dapagliflozin 10 mg: 160 Placebo: 161	eGFR: 45–59 mL/min/1.73 m^2^ (MDRD) UACR: ≥30 mg/g	65.3 years	Dapagliflozin: 53.3 ± 8.7 Placebo: 53.6 ± 10.6	Dapagliflozin: 23.5 (2.7–5,852.0) Placebo: 29.0 (3.8–8,474.0)	Change in UACR and eGFR from baseline at 24 weeks	SR and MA
Pollock *et al*.^12^ (2019)	Randomised, double-blind, multicentric, placebo-controlled trial	Dapagliflozin 10 mg OD	Placebo and dapagliflozin + saxagliptin	Standard pre-enrolment anti-diabetic and antihypertensive (ACEi, ARB) regimen given	Study duration: 24 weeks	Total: 461 Dapagliflozin 10 mg: 145 Placebo: 148	eGFR: 25–75 mL/min/1.73 m^2^ (MDRD) UACR: 30–3,500 mg/g	64.7 ± 8.6 years	Dapagliflozin: 50.2 ± 13.0 Placebo: 47.7 ± 13.5	Placebo: 257.5 (80–949) Dapagliflozin: 270.0 (69–751)	Change in UACR and eGFR from baseline at 24 weeks	SR and MA
Mosenzon *et al*.^10^ (2019)	Secondary exploratory analysis of randomised, double blind, placebo controlled trial	Dapagliflozin 10 mg OD	Placebo	Adjunct to standard care – pre-enrolment anti-diabetic regimen, ACEi, ARBs	Median follow-up years: 4 years	Total: 17,160 <90 ml/min/1.73 m^2^: 8,997 Dapagliflozin: 4,444 Placebo: 4,553	Not defined CrCl >60	eGFR 60–90: 66.2 ± 6.5 and eGFR <60 mL/min/1.73 m^2^: 67.3 ± 6.6	eGFR 60–90: 77.0 ± 8.5 eGFR <60: 51.4 ± 7.2	Overall: 13.1 (6.0–43.6)	Change in eGFR from baseline per year	SR and MA (<60 group alone)
Mosenzon *et al*.^11^ (2021)	Secondary exploratory analysis of randomised, double blind, placebo controlled trial	Dapagliflozin 10 mg OD	Placebo	Adjunct to standard care – pre-enrolment anti-diabetic regimen, ACEi, ARBs	Median follow-up years: 4 years	Total: 17,160 < 90 ml/min/1.73 m^2^: 8,997 Dapagliflozin: 4,444 Placebo: 4,553	Not defined CrCl >60	eGFR 60–90: 66.2 ± 6.5 and eGFR <60 mL/min/1.73 m^2^: 67.3 ± 6.6	eGFR 60–90: 77.0 ± 8.5 eGFR <60: 51.4 ± 7.2	Overall: 13.1 (6.0–43.6)	Change in UACR from baseline at 48 months	SR
Heerspink *et al*.^13^ (2021)	Randomised, double-blind, placebo-controlled, multicentre clinical trial.	Dapagliflozin 10 mg OD	Placebo	Stable maximum doses of ACEi and ARBs are given	Median follow-up years: 2.4 years	Total: 4,304 Diabetes: 2,906 Dapagliflozin and Diabetes: 1,455 Placebo and diabetes: 1,451	eGFR: 25–75 mL/min/1.73 m^2^ (CKD-EPI) UACR: 200–5,000 mg/g	62 ± 12.1 years	Both groups with diabetes: 43.8 ± 12.6	Both groups with diabetes: 1,016.5	Change in eGFR from baseline per year	SR and MA
Jongs *et al*.^14^ (2021)	Randomised, double-blind, placebo-controlled, multicentre clinical trial.	Dapagliflozin 10 mg OD	Placebo	Stable maximum doses of ACEi and ARBs are given	Median follow-up years: 2.4 years	Total: 4,304 Diabetes: 2,906 Dapagliflozin and Diabetes: 1,455 Placebo and diabetes: 1,451	eGFR: 25–75 mL/min/1.73 m^2^ (CKD-EPI) UACR: 200–5,000 mg/g	62 ± 12.1 years	Both groups with diabetes: 43.8 ± 12.6	Both groups with diabetes: 1,016.5	Change in UACR from baseline at 36 months	SR and MA
Huang *et al*.^6^ (2022)	Randomised, single centre, parallel group trial	Dapagliflozin 10 mg OD	Valsartan 80 mg BD	Standard anti-diabetic regimen followed	Study duration: 3 months	Total: 120 Dapagliflozin 10 mg: 60 Valsartan 80 mg: 60	eGFR: <60 mL/min/1.73 m^2^ (MDRD) UACR: ≥30 mg/g	56.21 ± 11.46 years	Not specified	Not specified	Change in eGFR from baseline at 12 weeks	SR

eGFR = estimated glomerular filtration rate; MDRD = modification of diet in renal disease; UACR = urine albumin-creatinine ratio; SD = standard deviation; SR = systematic review; MA = meta-analysis; OD = once daily; CKD-EPI = chronic kidney disease epidemiology collaboration.

**Table 2:** Summary of the findings of the studies included in the systematic review^6^–^14^

Mean change in eGFR from baseline				
Author and publication year	Outcome assessed	Number of participants	Results	Remarks
Kohan *et al*.^9^ (2014)	Mean change in eGFR from baseline at week 104. Reported as secondary objective.	Dapagliflozin: 85, Placebo: 84. At 104 weeks: Dapagliflozin: 50, Placebo: 42	Dapagliflozin: mean ± SE: −3.50 ± 1.02, Placebo: mean ± SE: −2.38 ± 1.01	Decrease in eGFR from baseline was more with dapagliflozin than with placebo after 104 weeks. Mean difference: −1.12 mL/min/1.73 m^2^ (95 % CI: −3.92–1.68)
Fioretto *et al*.^8^ (2018)	Mean change in eGFR from baseline at 24 weeks. Reported as safety endpoint.	Dapagliflozin: 160, Placebo: 161, At 24 weeks: Dapagliflozin: 150, Placebo: 145	Dapagliflozin: mean ± SE: −3.3 ± 1.25, Placebo: mean ± SE : −0.8 ± 1.31	Decrease in eGFR from baseline was more with dapagliflozin than with placebo after 24 weeks. Mean difference: −2.49 mL/min/1.73 m^2^ (95 % CI: −4.96–−0.02)
Pollock *et al*.^12^ (2019)	Mean change in eGFR from baseline at 24 weeks. Reported as safety endpoint.	Dapagliflozin: 145, Placebo: 148, At 24 weeks: Dapagliflozin: 131, Placebo: 134	Dapagliflozin: mean ± SE: −4 ± 0.80, Placebo: mean ± SE: −1.6 ± 0.80	Decrease in eGFR from baseline was more with dapagliflozin than with placebo after 24 weeks. Mean difference: −2.4 ml/min/1.73 m^2^ (95% CI: −4.2–−0.5; *P* = 0.01)
Mosenzon *et al*.^10^ (2019)	Mean change in eGFR from baseline at 4 years. Reported as pre-defined subgroup analysis of secondary composite outcome.	Dapagliflozin: 4,444 (60–90: 3,838; at 4 years: 2,686 <60: 606; at 4 years: 382), Placebo: 4,553 (60–90: 3,894; at 4 years: 2,631 <60: 659; at 4 years: 391)	60–90 eGFR: Dapagliflozin: mean ± SE: −8.18 ± 0.29, Placebo: mean ± SE: −9.81 ± 0.24. <60 eGFR: Dapagliflozin: mean ± SE: −2.45 ± 0.23, Placebo: mean ± SE: −4.27 ± 0.23	Decrease in eGFR was less with Dapagliflozin than with placebo in both the 60–90 and <60 eGFR groups. Mean difference: +1.63 and +1.82 mL/min/1.73 m^2^, respectively
Heerspink *et al*.^13^ (2021)	Mean change in eGFR from baseline per year. Reported as primary pre-specified outcome.	Dapagliflozin: 1,455, Placebo: 1,451. At 36 months: Dapagliflozin: 113, Placebo: 108	Dapagliflozin: mean ± SE: −2.84 ± 0.14, Placebo: mean ± SE: −4.01 ± 0.14	Compared to placebo, dapagliflozin attenuated the loss of kidney function more. Mean difference: + 1·18 mL/min/1.73 m^2^ per year (95% CI: 0·79–1·56)
Huang *et al*.^6^ (2022)	Mean change in eGFR from baseline at 12 weeks. Reported as secondary outcome.	Dapagliflozin: 60, Valsartan: 60	Dapagliflozin: Baseline:111.17 ± 29.22. At 12 weeks: 113.01 ± 26.66. Valsartan: Baseline: 110.08 ± 27.64 At 12 weeks: 111.79 ± 24.72	eGFR increased by + 1.84 mL/min/1.73 m2 in the dapagliflozin group and by + 1.71 mL/min/1.73 m2 in the valsartan group. Mean difference: 0.13 mL/min/1.73 m^2^ (*P* >0.05)
**Mean change in UACR from baseline**				
Fioretto *et al*.^7^ (2016)	Mean % change in UACR from baseline at 104 weeks. Reported as exploratory endpoint.	Dapagliflozin: 56, Placebo: 57. At week 104: Dapagliflozin: 29, Placebo: 25	Dapagliflozin: mean ± SE: −43.9 ± 15.6, Placebo: mean ± SE: 31 ± 39.1	Placebo-corrected UACR reductions (95% CI) of −57.2% (−77.1–−20.1%) occurred in the dapagliflozin group.
Fioretto *et al*.^8^ (2018)	Mean % change in UACR from baseline at 24 weeks. Reported as exploratory endpoint.	Dapagliflozin: 160, Placebo: 161. At 24 weeks: Dapagliflozin: 60, Placebo: 69	Dapagliflozin: mean ± SE: −43.7 ± 14.8, Placebo: mean ± SE: −34.6 ± 16.2	Dapagliflozin reduced the mean percentage changes in UACR from baseline at week 24. Mean difference: −9.0% (95% CI: −52.19–33.99%; *P* = 0.4)
Pollock *et al*.^12^ (2019)	Mean % change in UACR from baseline at 24 weeks. Reported as primary efficacy endpoint.	Dapagliflozin: 145, Placebo: 148. At 24 weeks: Dapagliflozin: 132, Placebo: 132	Dapagliflozin: mean ± SE: −22.92 ± 7.24, Placebo: mean ± SE: −1.7 ± 9.09	Dapagliflozin significantly reduced UACR. Difference in mean change in UACR from baseline: −21·0% (−34.1–−5.2%; *P* = 0.011]
Mosenzon *et al*.^11^ (2021)	Mean change in UACR from baseline at 48 months. Reported as pre-defined subgroup analysis of secondary composite outcome.	Dapagliflozin: 4,444 (60–90: 3,838; at 4 years: 2,612 <60: 606; at 4 years: 367), Placebo: 4,553 (60–90: 3,894; at 4 years: 2,552 <60: 659; at 4 years: 376)	60–90 eGFR: Dapagliflozin: mean UACR mg/g Baseline: 19.89; at 48 months: 23.23, Placebo: mean UACR mg/g: Baseline: 20.32; at 48 months: 27.20. <60 eGFR: Dapagliflozin: mean UACR mg/g Baseline: 32.6; At 48 months: 40.82 Placebo: mean UACR mg/g: Baseline: 36.16; at 48 months: 60.27	Dapagliflozin treatment caused a significant reduction in UACR (*P* <0.001) compared to placebo in both eGFR groups at 6 months, and this was sustained throughout the 4-year duration of the study.
Jongs *et al*.^14^ (2021)	Mean % change in UACR from baseline at 36 months. Reported as pre-specified exploratory outcome.	Dapagliflozin: 1,455, Placebo: 1,451. At 36 months: Dapagliflozin: 159, Placebo: 158	Dapagliflozin: mean ± SE: −42 ± 3.72, Placebo: mean ± SE: −17 ± 5.54	Relative to placebo, treatment with dapagliflozin resulted in a mean percentage change in UACR of −25% (95% CI: −38·03–−11.97; *P* <0·0001) at 36 months.

eGFR: estimated glomerular filtration rate; SE: standard error; CI: confidence interval; UACR: urine albumin-creatinine ratio.
